# Characterization of Novel Reassortant Influenza A (H5N2) Viruses Isolated from Poultry in Eastern China, 2015

**DOI:** 10.3389/fmicb.2017.00741

**Published:** 2017-04-25

**Authors:** Haibo Wu, Rufeng Lu, Xiuming Peng, Xiaorong Peng, Linfang Cheng, Fumin Liu, Nanping Wu

**Affiliations:** ^1^Collaborative Innovation Center for Diagnosis and Treatment of Infectious Diseases, State Key Laboratory for Diagnosis and Treatment of Infectious Diseases, the First Affiliated Hospital, School of Medicine, Zhejiang UniversityHangzhou, China; ^2^Department of Emergency, the First Affiliated Hospital of Zhejiang Chinese Medical UniversityHangzhou, China

**Keywords:** avian influenza viruses, subtype H5N2, poultry, reassortant, eastern China

## Abstract

Recently, novel variants of H5 highly pathogenic avian influenza viruses (AIVs) have been frequently isolated from poultry and wild birds in Asia, Europe and North America. Live poultry markets (LPMs) play an important role in the dissemination of influenza viruses. Four H5N2 AIVs were isolated from poultry during surveillance of AIVs in LPMs in Eastern China, in 2015. Whole-genome sequencing, combined with phylogenetic and antigenic analyses were performed to characterize these viruses. These H5N2 viruses had undergone extensive reassortment resulting in two genetic groups of viruses in poultry. These viruses exhibited slightly pathogenicity in mice, and replicated without prior adaptation. The continued circulation of these novel H5N2 viruses may represent a threat to human health.

## Introduction

The emergence of H5N1 highly pathogenic avian influenza (HPAI) has caused severe epidemics among poultry and has resulted in large-scale economic losses worldwide (Li et al., 2004, 2010). As of 21 November 2016, 856 human cases of H5N1 avian influenza virus (AIV) infection had been reported to the WHO; 452 (52.8%) cases were fatal (WHO, 2016). In 2014, a novel H5N6 HPAI virus was reported in China, and linked to the death of a man, believed to be the world’s first human infected with an H5N6 virus (WHO, 2014). In the same year, in China and Laos, a large population of poultry was infected with an H5N6 HPAI virus and resulted in poultry deaths (OIE, 2014; Wong et al., 2015). Since 2013, novel variants of H5N8 HPAI viruses have been isolated from poultry and wild birds in China (Wu et al., 2014b) and South Korea (Lee et al., 2014) in East Asia. These variants are now co-circulating in many European countries (Adlhoch et al., 2014; Bouwstra et al., 2015) and North America (Jhung and Nelson, 2015; Lee et al., 2015).

In Asia, H5N2 AIVs have previously been found mainly in migratory birds, while more recently, transmission to chickens and ducks has been reported (Cheng et al., 2010; Zhao et al., 2012; Wu et al., 2014a). Previous studies showed that people who are in frequent contact with infected animals can be infected by the H5N2 viruses (Ogata et al., 2008; Yamazaki et al., 2009; Shafir et al., 2012; Wu et al., 2014c). A large number of novel HPAI H5 virus infections have occurred in the human population and a lack of pre-existing immunity to H5 AIVs poses a potential public health risk.

Previous studies have shown that aquatic birds, including ducks, represent the major natural reservoir of AIVs (Olsen et al., 2006). Furthermore, chickens have the molecular characteristics of a potential intermediate host for AIVs in the transmission to humans and could generate new influenza viruses with pandemic potential (Guo et al., 2007; Gambaryan et al., 2008). Live poultry markets (LPMs) are considered to be a major source of AIV dissemination, and sites for potential AIV reassortment and cross-species transfer. Active surveillance of AIVs in ducks and chickens from LPMs should be implemented as an early warning system for avian influenza outbreaks (Liu et al., 2003; Chen et al., 2013).

During the surveillance for avian influenza in LPMs in Zhejiang Province, Eastern China, in 2015, four H5N2 AIVs were isolated from apparently healthy poultry. Whole-genome sequencing, combined with phylogenetic and antigenic analyses indicated the existence of reassortment between different AIV subtypes from avian species. The continued circulation of these H5N2 viruses may pose a potential threat to human health.

## Materials and Methods

### Ethics Statement

The female 6-week-old specific pathogen-free BALB/c mice used in this study were purchased from Shanghai Laboratory Animal Center, Chinese Academy of Sciences, Shanghai, China. These animal studies were approved by the First Affiliated Hospital, School of Medicine, Zhejiang University (No. 2015-015). All of the experiments with the AIVs were performed in a Level 3 Biosafety laboratory (registration No. CNAS BL0022, the First Affiliated Hospital, School of Medicine, Zhejiang University).

### Virus Isolation

Cloacal swabs (*n* = 1,250) were collected from apparently healthy poultry [chickens (*n* = 900), ducks (*n* = 300), and pigeons (*n* = 50)] in LPMs (*n* = 6) in Hangzhou, Zhejiang Province, Eastern China, from January 2015 to December 2015. Each swab was eluted with 2.0 mL phosphate-buffered saline (PBS) containing 0.2% bovine serum albumin (BSA), 4 × 10^6^ U/L penicillin G, and 400 mg/L streptomycin sulfate. A 0.22-μm filter was used to decontaminate the samples, which were then inoculated into the allantoic cavities of 9-day-old specific pathogen-free embryonated eggs as described previously (Wu et al., 2012). After incubation at 37°C for 72 h, the allantoic fluid was harvested and viral titers were determined by hemagglutination (HA) assay using a standard method (Klimov et al., 2012). Briefly, 1% chicken red blood cells (specific pathogen-free) were prepared. HA titers were then determined by adding 50 μL of the 1% chicken red blood cells in PBS to 50 μL of a twofold serial dilution of virus in 96 ‘V’-well microtiter plates. The microtiter plates were incubated for 30 min at 25°C. HA titers were defined as the reciprocal values of the highest dilutions that caused complete HA.

### RNA Extraction and PCR Amplification

RNA was extracted from the HA-positive allantoic fluid samples using TRIzol (Life Technologies, USA) according to the manufacturer’s instructions. Reverse transcription was performed using the Uni12 primer: 5′–AGCAAAAGCAGG–3′. Reverse transcriptase-polymerase chain reaction (RT-PCR) was conducted using a One-Step RNA PCR Kit (TaKaRa, China). All segments were amplified with previously described segment–specific primers (Hoffmann et al., 2001). The PCR products were purified using the Agarose Gel DNA Fragment Recovery Kit Ver. 2.0 (TaKaRa).

### Sequencing and Phylogenetic Analysis

The amplified fragments were sequenced using a Big Dye Terminator V.3.0 Cycle Sequencing Ready Reaction kit (ABI, Foster City, CA, USA) according to the manufacturer’s instructions. All eight gene segments [polymerase basic protein 2 (PB2), polymerase basic protein 1 (PB1), polymerase acidic protein (PA), HA, nucleocapsid protein (NP), NA, matrix protein (M), and non-structural protein (NS)] of the AIV field isolates were sequenced and compared with those of the reference viruses. The classical reference viruses were selected based on previous reports (Gu et al., 2011; Lee et al., 2014; Wu et al., 2014a; Wong et al., 2015), and the reference sequences of the strains used in this study were obtained from the Influenza Virus Resource^1^. The sequences were analyzed using BioEdit version 7.0.9.0 DNA software. Phylogenetic trees were constructed using molecular evolutionary genetics analysis (MEGA) software version 6.0, applying the maximum likelihood method and the Tamura–Nei model with bootstrap analysis (1,000 replicates) (Tamura et al., 2013). The nucleotide sequences were deposited into GenBank under the accession numbers: KX602675–706.

### Antigenic Analysis

The antigenic characteristics of these H5N2 viruses were analyzed with the hemagglutination inhibition (HI) test as described elsewhere (Chang et al., 2014). Mouse antisera against A/duck/Zhejiang/224/2011(H5N1), A/duck/Zhejiang/6DK19/2013(H5N2), A/chicken/Zhejiang/6C2/2013(H5N6), and A/duck/Zhejiang/W24/2013(H5N8), were provided by our laboratory. These viruses were all isolated in recent years from LPMs in Zhejiang Province as described previously (Hai-bo et al., 2012; Wu et al., 2014a,b, 2015a). Chicken antisera against the new H5 inactivated Re-8 vaccine (purchased from Harbin Weike Biotechnology Development Company) which has been used in the field in China to prevent the HPAI H5 variants in clade 2.3.4.4 (Zeng et al., 2016), was also analyzed in this study.

### Animal Study

To evaluate virus pathogenicity and replication potential in mammalian hosts, fifteen 6-week-old female BALB/c mice were inoculated intranasally with 10^6.0^ 50% egg infective dose (EID_50_) of virus in 50 μL PBS. At 3, 6, and 9 days post-inoculation, three mice were sacrificed and their lungs, brain, heart, liver, kidneys, and spleen were collected to determine viral distribution in the tissues based on virus titration in embryonated chicken eggs. The organs were homogenized in 1 mL of cold PBS, and centrifuged at 2,500 × *g* for 10 min. Embryonated chicken eggs were used to determine the EID_50_ values of the supernatants using the method described by Reed and Muench (Reed and Muench, 1938). The survival rate and weight-loss were monitored everyday in the remaining six mice over 14 days following inoculation as described previously (Hai-bo et al., 2012). A group of mock-infected mice was included as a control. The animal studies were performed in accordance with the recommendations of the Office International des Epizooties (OIE) (Pearson, 2003).

### Histological and Immunohistological Analysis of Mouse Lung Sections

Lung tissues samples from virus-inoculated mice (portions from the lungs used for virus titration) were fixed in 10% neutral buffered formalin for at least 24 h before processing. The tissues were embedded in paraffin by standard tissue processing procedures. Sections (thickness, 4 μm) were cut and fixed on glass slides and stained with hematoxylin and eosin (H&E).

Immunohistochemical staining to detect nucleoprotein antigens in the lungs was performed by incubation with a monoclonal antibody against the influenza A virus nucleoprotein (1:100 dilution, Cat. No.SAB 5300169, Sigma) at 4°C overnight. The sections were washed three times with PBS and then incubated with HRP-conjugated goat anti-mouse secondary antibody (1:3,000 dilution, Sigma). The sections were developed with 3–3′ diaminobenzidine and examined with a light microscope as described previously (Wu et al., 2015b).

## Results

### Virus Isolation

Swab samples were collected in LPMs in the Yuhang and Xihu districts of Hangzhou City, the capital of Zhejiang Province; 119 strains of AIVs were isolated. Of these, 93 were isolated from 900 chicken samples (isolation rate: 10.3%); 21 were isolated from 300 duck samples (7.0%); and five were isolated from 50 pigeon samples (10.0%). On average, AIVs were isolated from 9.5% (119/1,250) of the swab samples. These 119 AIVs included seven HA subtypes (H1, H3, H4, H5, H6, H7, and H9), four NA subtypes (N2, N3, N6, and N9), and 11 AIVs subtypes. The epidemiologic information for all 119 AIVs is provided in **Table 1**.

**Table 1:** General overview of the avian influenza viruses collected from poultry in live poultry markets in Zhejiang Province from January 2015 to December 2015.

HA subtypes (%)	AIV subtypes	Total number of strains	Species (number of strains)
H1 (4/119, 3.5%)	H1N2	2	Chicken (2)
	H1N3	2	Chicken (2)
H3 (51/119, 42.9%)	H3N2	12	Chicken (10), duck (2)
	H3N6	39	Chicken (34), duck (1), pigeon (4)
H4 (2/119, 1.7%)	H4N2	2	Duck (2)
H5 (8/119, 6.7%)	H5N2	4	Chicken (3), duck (1)
	H5N6	4	Chicken (4)
H6 (29/119, 24.4%)	H6N6	29	Chicken (25), duck (4)
H7 (10/119, 8.4%)	H7N3	2	Chicken (2)
	H7N9	8	Chicken (4), duck (4)
H9 (15/119, 12.6%)	H9N2	15	Chicken (7), duck (7), pigeon (1)
**Total**	11 subtypes	119 strains	Chicken (93/119, 78.2%), Duck (21/119, 17.6%), Pigeon (5/119, 4.2%)

### Phylogenetic Analysis of H5N2 AIV

**FIGURE 1:**
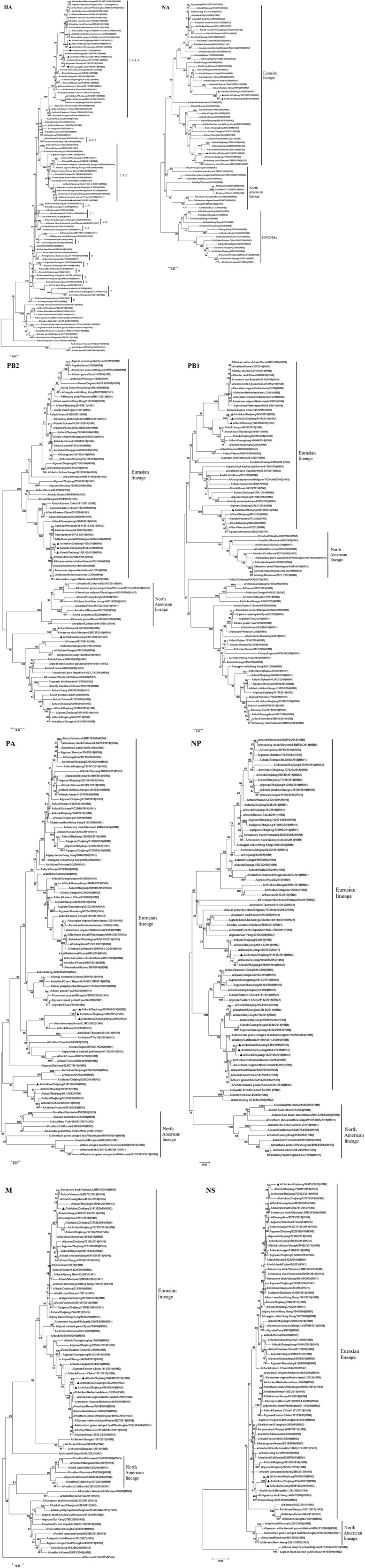
**Phylogenetic analysis of the HA (positions 1–1,704), NA (positions 40–1,341), PB2 (positions 1–2,280), PB1 (positions 16–2,268), PA (positions 1–2,151), NP (positions 1–1,436), M (positions 29–923), and NS (positions 28–768) of H5N2 avian influenza viruses compared to reference influenza viruses obtained from the Influenza Virus Resource (http://www.ncbi.nlm.nih.gov).** The phylogenetic tree was created by the maximum likelihood method and bootstrapped with 1,000 replicates using the MEGA software version 6.0. Chinese avian influenza viruses from poultry in this study are highlighted by triangles, and the novel 2014–2015 H5N6 influenza virus, which caused human infection, is indicated by a dot. Scale bar represents the distance unit between sequence pairs.

Phylogenetic analysis of all eight gene segments, PB2, PB1, PA, HA, NP, NA, M, and NS, showed that these H5N2 strains clustered in the AIV Eurasian lineage (**Figure 1**).

The sequences of the HA and NA genes of the H5N2 viruses identified in this study indicated that two different genetic groups were co-circulating in Zhejiang Province, in 2015. Phylogenetic analysis of the HA genes showed that these H5N2 AIVs belonged to clade 2.3.4.4. The HA genes of A/chicken/Zhejiang/7450/2015 (H5N2; ZJ-7450), A/chicken/Zhejiang/81643/2015(H5N2; ZJ-81643) and A/duck/Zhejiang/1026109/2015(H5N2; ZJ-1026109; Group I), were most closely related to many novel H5N8 and H5N2 AIVs circulating in birds from South Korea, Japan, Europe, and North America since 2014. The HA gene of A/chicken/Zhejiang/514135/2015(H5N2; ZJ-514135; Group II) showed the highest nucleotide similarity to Chinese isolates from poultry, originating from the same ancestors of the novel 2014 H5N6 virus responsible for human infection in Southern China (Bi et al., 2015).

The NA gene phylogeny indicated that the NA genes of Group I were most closely related to many H5N2 AIVs isolated from Eastern China since 2011, while the NA genes of Group II showed the highest nucleotide similarity to other AIV subtypes (such as H6N2 and H3N2) originating from Eastern Asia.

The overall gene sequence homologies between these H5N2 strains and the AIV reference strains for HA and NA were similar; therefore, ZJ-1026109 and ZJ-514135 were selected as representative isolates for more in-depth analysis. The percentage sequence homology for each gene segment in ZJ-1026109 and ZJ-514135 compared with their closest genetic relative is shown in **Table 2**.

**Table 2:** Sequence similarities of whole genomes of the H5N2 isolates compared to nucleotide sequences available in the GenBank database.

Segment	Position	Virus with the highest percentage of nucleotide identity	GenBank accession number	Homology (%)
**Isolate 1: ZJ-1026109**				
PB2	1-2280	A/baikal teal/Korea/1449/2014(H5N8)	KJ756568	98
PB1	1-2274	A/duck/Jiangsu/k1203/2010(H5N8)	JQ973692	98
PA	1-2151	A/mallard/Jiangxi/8264/2004(H6N2)	HM145335	97
HA	1-1695	A/baikal teal/Korea/2417/2014(H5N8)	KJ756628	99
NP	1-1490	A/baikal teal/Korea/1456/2014(H5N8)	KJ756647	98
NA	1-1410	A/goose/Eastern China/1112/2011(H5N2)	JQ041410	97
M	1-924	A/Korean native chicken/Korea/H257/2014(H5N8)	KJ509127	99
NS	1-838	A/duck/Jiang Xi/6146/2003(H5N3)	EF597369	98
**Isolate 2: ZJ-514135**				
PB2	1-2280	A/duck/Vietnam/LBM760/2014(H5N6)	LC028344	98
PB1	1-2274	A/duck/Zhejiang/4812/2013(H3N8)	KF357801	98
PA	1-2151	A/duck/Zhejiang/0611-17/2011(H1N3)	JN605395	97
HA	1-1695	A/duck/Guangzhou/41227/2014(H5N6)	KP765796	99
NP	1-1490	A/duck/Zhejiang/925096/2014(H4N2)	KT589238	98
NA	1-1413	A/duck/Zhejiang/727042/2014(H6N2)	KT423163	99
M	1-924	A/duck/Guangzhou/41227/2014(H5N6)	KP765799	99
NS	1-838	A/duck/Guangzhou/41227/2014(H5N6)	KP765800	98

BLAST analysis of the homology with sequences in the GenBank database showed that the PB2, HA, NP, and M gene segments of ZJ-1026109 were most closely related to 2014 Korean H5N8 AIVs, while the others gene segments (PB1, NA, PA, and NS) were most closely related to certain Chinese AIVs (such as H5N2, H5N3, and H6N2), which originated from migratory birds and poultry. All the gene segments of ZJ-514135 were most closely related to certain poultry AIVs (such as H5N6, H6N2, H4N2, and H3N8) isolated from Eastern Asia (**Table 2**). And the internal genes were not closely related to H9N2-like segments that were found in human-infecting HPAI H5N6, H7N9, and H10N8 viruses in China. This analysis indicated that ZJ-1026109 and ZJ-514135 are reassortant viruses. Furthermore, our results suggest that these H5N2 viruses also underwent reassortment with novel H5N8 viruses and other subtypes (H5, H6, H3, and H4) of viruses isolated from chickens and ducks in Eastern China. These results also provide further evidence of the active evolution of H5 AIVs in Eastern China.

### Molecular Characterization of H5N2 AIV

Having established the genetic context of these H5N2 AIVs, we characterized these strains at the molecular level. It is widely known that the addition of amino acids such as Arg (R) and Lys (K) at HA cleavage sites in the H5 subtype AIVs can convert low-pathogenic avian influenza into HPAI (Horimoto et al., 1995). Based on the deduced amino acid sequences of the HA genes, the HA cleavage site pattern of these H5N2 strains is PLRERRRKR/GL, suggesting that these H5N2 strains are HPAI viruses.

**FIGURE 2:**
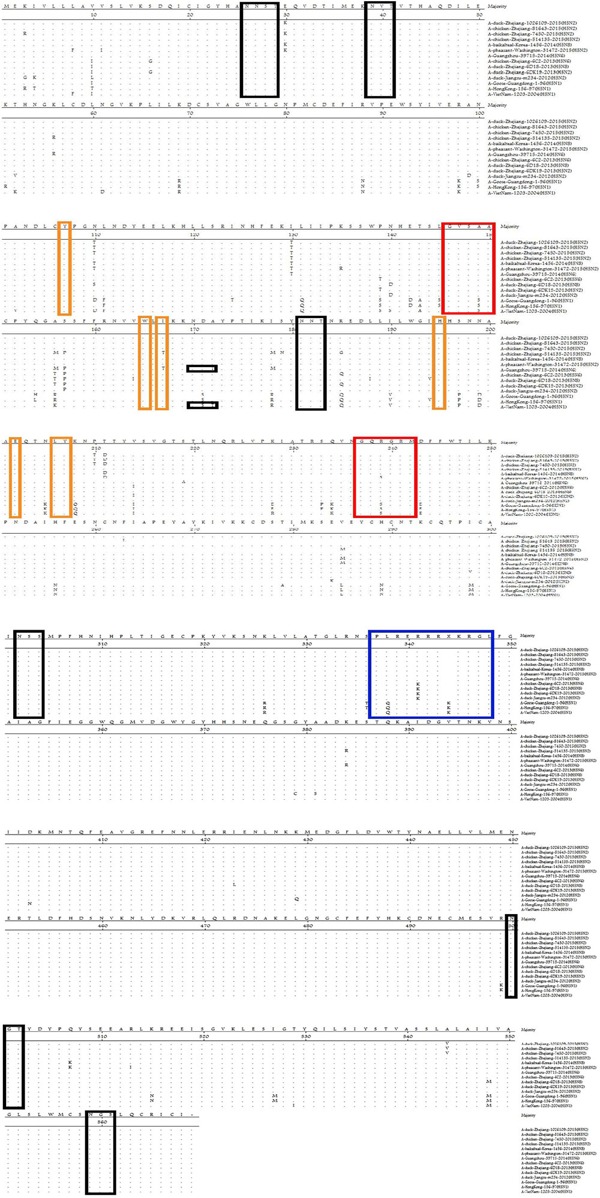
**Comparison of deduced amino acid sequences of HA using the MegAlign program.** Identical amino acids are shown as dots. The blue box represents the HA sequence at the cleavage site, black boxes represent potential *N*-linked glycosylation sites, and red/yellow boxes indicate residues involved in the receptor-binding site.

We also analyzed the amino acids [107(Y), 165(W), 167(I/T), 195(H), 202(E), 202(L), and 203(Y)] at receptor-binding sites (RBSs) of HA (**Figure 2**). The amino acids of these H5N2 viruses at positions 236–241 and 146–150 were “NGQRGR” and “GVSAA,” respectively. The RBSs of the H5N2 viruses contained Gln226 and Gly228 (H3 numbering system), which suggested preferential binding to avian-like receptors (Hai-bo et al., 2012; Wu et al., 2014b).

Hemagglutination glycosylation is associated with virulence and viral affinity for the influenza virus receptor (Iqbal et al., 2012; Zhang et al., 2013). Seven potential N-linked glycosylation sites in HA (26, 27, 39, 181, 302, 500, and 559; **Figure 2**) and eight in NA (42, 61, 69, 70, 86, 146, 200, and 234) were detected in the strains identified in this study. The specific polypeptide for N-linked glycosylation is defined as (Asn–X–Ser/Thr), where X can be any amino acid, except Pro (P), or Asp (D) (Helenius and Aebi, 2004).

The Glu627Lys, Asp701Asn, Ser714Arg, Glu158Gly, and Thr271Ala substitutions in the PB2 protein, and Thr97Ile substitution in the PA protein, are associated with the host range and confer increased virulence of H5 viruses in mice (Shinya et al., 2004; Song et al., 2009; Bussey et al., 2010; Zhou et al., 2011; Schat et al., 2012; Czudai-Matwich et al., 2014). None of these substitutions were observed in the PB2 or PA from the H5N2 viruses analyzed in this study (**Table 3**).

**Table 3:** Features of critical amino acid residues in the novel reassortant H5N2 avian influenza viruses isolated from poultry in LPMs in Zhejiang Province, Eastern China.

	ZJ-1026109	ZJ-7450	ZJ-81643	ZJ-514135	Function
HA	Gly	Gly	Gly	Gly	Gly186Val increases binding affinity for α-2,6-linked sialic acid receptor.
	Gln	Gln	Gln	Gln	Gln226Leu increases binding affinity for α-2,6-linked sialic acid receptor.
NA	No	No	No	No	A short-stalk increases virulence in mice.
	Glu	Glu	Glu	Glu	Glu119Val substitutions are the molecular markers of oseltamivir-resistance in H5N2.
PB2	Glu	Glu	Glu	Glu	Glu627Lys results in mammalian host adaptation.
	Asp	Asp	Asp	Asp	Asp701Asn enhances transmission in guinea pigs.
	Ser	Ser	Ser	Ser	Ser714Arg promotes mammalian host adaptation.
	Thr	Thr	Thr	Thr	Thr271Ala enhances polymerase activity in mammalian host cells.
	Glu	Glu	Glu	Glu	Glu158Gly enhances viral replication rates in mammalian host cells.
	Gln	Gln	Gln	Gln	Gln591 Lys supports virus replication in mammals.
M2	Asn	Asn	Asn	Ser	Ser31Asn causes resistance to adamantanes.
	Val	Val	Val	Val	Val27Ala causes resistance to adamantanes.
NS1	Ser	Ser	Ser	Ser	Pro42Ser increases pathogenicity in mice.
	No	No	No	Yes	Deletion of amino acids (aa) at position 80–84 indicate adaptation to avian species.
PB1-F2	90 aa in	90 aa in	90 aa in	90 aa in	Increases pathogenicity in mice.
	length	length	length	length	
	Ser	Ser	Ser	Ser	Asn66Ser increases pathogenicity in mice.

A deletion of five amino acids “TIASV” (position 80–84) in the NS1 proteins was observed in the ZJ-514135; however, it was not observed in the remaining three H5N2 viruses. All H5N2 viruses characterized in this study contained the Pro42Ser substitution in NS1, which is associated with increased virulence in mice (Jiao et al., 2008).

NA inhibitors are effective antiviral drugs for treatment of influenza virus infections, and the His275Tyr and Glu119Val substitutions are the molecular markers of oseltamivir-resistance in H5N2 viruses. Previous studies predicted lower viability among resistant mutant viruses compared with sensitive strains (Aoki et al., 2007; Zaraket et al., 2010; Achenbach and Bowen, 2013). Neither of these substitutions was observed in the NA of the H5N2 AIVs. In recent years, the Val27Ala and Ser31Asn substitutions in the M2 protein, which are associated with amantadine-resistance (Deyde et al., 2007; Tosh et al., 2014), have been observed frequently in H5N1 AIVs in China (He et al., 2008; Dong et al., 2014). In this study, ZJ-7450, ZJ-81643, and ZJ-1026109, had the Ser31Asn substitution in the M2 protein, while this substitution was not observed in ZJ-514135. None of the H5N2 viruses had the Val27Ala substitution in the M2.

### Antigenic Analysis of H5N2 AIV

To evaluate the antigenic characteristics of these H5N2 viruses, we used the HI assay to compare cross–reactivity with other H5 (H5N1, H5N2, H5N6, and H5N8) viruses isolated from chickens and ducks in Zhejiang Province in recent years and Re-8 (**Table 4**). The results showed these four H5N2 strains were antigenically most distinct from A/duck/Zhejiang/224/2011(H5N1), whereas they cross-reacted more closely with the other three H5 viruses, A/duck/Zhejiang/6DK19/2013(H5N2), A/chicken/Zhejiang/6C2/2013(H5N6), A/duck/Zhejiang/W24/2013(H5N8), and Re-8.

**Table 4:** Antigenic analysis of the novel reassortant H5N2 avian influenza viruses according to the hemagglutination inhibition test.

Polyclonal antisera titers to:	Novel reassortant H5N2 virus			
	ZJ-1026109	ZJ-7450	ZJ-81643	ZJ-514135
A/duck/Zhejiang/224/2011(H5N1)	16	16	16	16
A/duck/Zhejiang/6DK19/2013(H5N2)	32	32	32	64
A/chicken/Zhejiang/6C2/2013(H5N6)	32	32	32	32
A/duck/Zhejiang/W24/2013(H5N8)	64	64	64	64
Re-8 vaccine	64	64	64	64

### Pathogenicity in Mice

**FIGURE 3:**
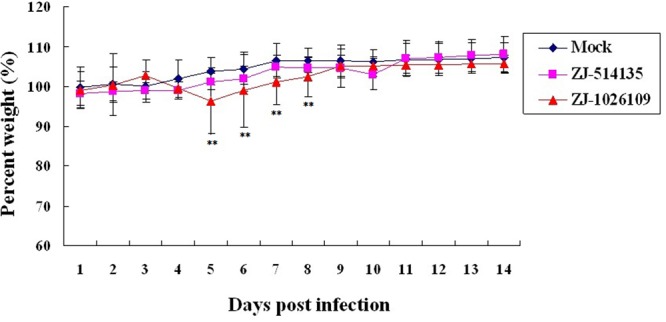
**Weight variation of BALB/c mice infected with the H5N2 avian influenza viruses.** Each mouse in a group was infected intranasally with 10^6.0^ EID_50_ of each virus. The survival rates and body weight of mice were measured daily from the date of challenge to 14 days after challenge (^∗∗^*P* < 0.05; one-way ANOVA).

To evaluate the pathogenicity and replication potential of the ZJ-1026109 and ZJ-514135 viruses in mammalian hosts, BALB/c mice were infected intranasally with 10^6.0^ EID_50_ of each virus. The mice infected with the ZJ-514135 virus exhibited only slight weight-loss. While the mice infected with the ZJ-1026109 virus exhibited weight-loss beginning on 2 dpi, and was significantly weight differences compared to mock from 5 to 8 dpi (*P* < 0.05; one-way ANOVA test; **Figure 3**). Both of the viruses were able to replicate without prior adaptation. During the 14 dpi with the ZJ-1026109 and ZJ-514135 viruses, survival rates of 83.3% (5/6) and 100% (6/6), respectively, were observed (**Table 5**). Taken together, these results indicate that these H5N2 viruses are slightly virulent in mice.

**Table 5:** Pathogenicity of the novel reassortant H5N2 avian influenza viruses isolated from poultry in LPMs in Zhejiang Province, Eastern China.

Virus	No. of survivors/no. tested	HI titer (log2)	Virus replication in experimentally infected mice Virus titers in organs of mice (log_10_ EID_50/_mL)			
			Tissue	Day 3	Day 6	Day 9
ZJ-1026109	5/6	6.17 ± 0.75	Lung	3.67 ± 0.58	4.67 ± 0.58	2.67 ± 0.58
			Brain	1.33 ± 0.58	1.67 ± 0.58	ND
			Heart	1.33 ± 0.58	1.67 ± 0.58	1.00 ± 0
			Liver	1.67 ± 0.58	1.83 ± 0.29	1.67 ± 0.58
			Kidney	ND	ND	ND
			Spleen	ND	1.00 ± 0	ND
ZJ-514135	6/6	5.67 ± 0.82	Lung	3.33 ± 0.58	3.67 ± 0.58	2.33 ± 0.58
			Brain	1.00 ± 0	1.33 ± 0.58	ND
			Heart	1.00 ± 0	1.50 ± 0.50	1.00 ± 0
			Liver	1.83 ± 0.29	2.00 ± 0	1.67 ± 0.58
			Kidney	ND	ND	ND
			Spleen	ND	1.00 ± 0	ND

*Fifteen mice were inoculated intranasally with 10^*6*.*0*^ EID_*50*_ of each H5N2 virus. Six mice from each group were observed for survival within 14 days. The remaining nine mice from each experimental group were sacrificed on days 3, 6, and 9, and organs (lung, brain, heart, kidney, spleen, and liver) were collected for virus titration in embryonated chicken eggs using the method described by Reed and Muench. Data represent the means ± SD of the values obtained from three infected mice. Sera were harvested at 14 days after infection from the six mice that survived. Seroconversion was confirmed by the HI test. ND, Not detected.*

**FIGURE 4:**
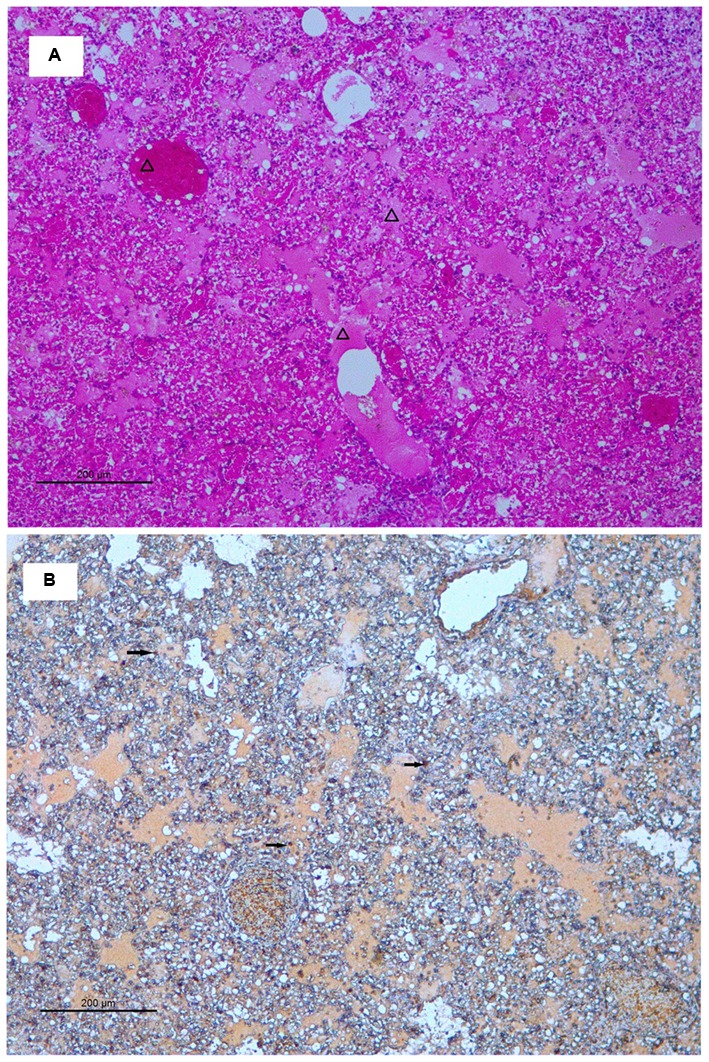
**Histology and immunohistochemistry of mice infected with ZJ-1026109 at 6 days post-infection. (A)** Histology of lung sections from inoculated mice stained with hematoxylin and eosin. **(B)** Immunohistochemical detection of viral nucleoprotein in lungs from inoculated mice. Triangles indicate alveolar edema containing fibrin, erythrocytes, and inflammatory cells. Arrows indicate positively stained lung alveolar epithelial cells.

Histopathological analyses showed that there were no significant pathological changes in the lung tissue of the mice infected with ZJ-514135; while, 3 days post-inoculation with ZJ-1026109, multifocal mild or moderate interstitial inflammatory hyperaemia and exudative pathological changes were observed in the lung. By 6 days post-inoculation, the lesions in the lung tissue had increased in size, and multiple patchy lesions had fused. More severe diffuse pneumonia, with alveolar edema containing fibrin, erythrocytes and inflammatory cells was also observed (**Figure 4A**).

Immunohistochemical analysis was also performed to detect the presence of H5N2 AIV-infected cells in the tissues (including bronchial epithelial cells and alveolar epithelial cells) of ZJ-1026109 infected mice at 6 days post-inoculation (**Figure 4B**). The H5N2 viruses replicated in both bronchial and alveolar epithelial cells. In addition, the H5N2 viruses caused severe lung injury in infected mice, which was manifested as congestion and bleeding.

## Discussion

The H5N2 viruses can infect a wide variety of species, including both avian species (migratory birds and poultry) (Gaidet et al., 2008; Kim et al., 2010; Snoeck et al., 2011) and mammalian species. To date, the Influenza Virus Resource contains three H5N2 virus isolates, A/swine/Korea/C12/2008(H5N2), A/swine/Korea/C13/2008(H5N2), and A/dog/Shandong/JT01/2009(H5N2), which were isolated from mammalian species (pigs and dogs) in Asia (Lee et al., 2009; Song et al., 2013). Although H5N2 viruses have not been detected in humans, previous studies have shown that the H5N2 subtype AIVs can infect people who are in frequent contact with infected birds and pigs, such as live poultry vendors, poultry farmers, and veterinarians (Ogata et al., 2008; Di Trani et al., 2012; Shafir et al., 2012; Wu et al., 2014c; Huang et al., 2015). These reports indicate the ability of H5N2 subtype AIVs to cross the species barrier to infect humans.

As a conventional animal model, mice are often used to study the pathogenesis of influenza A viruses, including avian strains (Brown, 1990; Belser et al., 2013). Previously, many studies have reported variable pathogenicity of different H5N2 strains in mice (Zhao et al., 2012; Wu et al., 2014a), and the novel H5N2 virus has been shown to cause severe disease in mice (Pulit-Penaloza et al., 2015). In the present study, our results suggest that these novel H5N2 AIVs did not cause lethal infections in mice, but possessed the ability to replicate well in the lungs of the mice without prior adaptation. The continued circulation of these H5N2 viruses may represent a threat to human health.

Phylogenetic analyses in previous reports showed that the H5 viruses undergo frequent reassortment, with multiple virus subtypes (such as H3, H6, H9, and H11) found in the gene pool in apparently healthy poultry. As a result, multiple novel variants have emerged from different lineages in poultry (Gu et al., 2012, 2014; Hai-bo et al., 2012; Zhang et al., 2012; Zhao et al., 2012; Lam et al., 2013; Wu et al., 2014a; Pasick et al., 2015). These novel H5N8 and H5N6 subtype AIVs have been present in Eastern China for several years, with the more recent spread to other countries caused by migration of wild birds (Wu et al., 2014b, 2015a; Bouwstra et al., 2015; Verhagen et al., 2015). The HPAI H5N8 virus responsible for outbreaks in Dutch poultry farms in 2014 were descended from an H5N8 virus that circulated around Asia in 2009, subsequently spreading to South Korea and Japan and finally also to Europe via migrating waterfowl (Bouwstra et al., 2015; Verhagen et al., 2015). The H5N8 virus isolated in the UK in 2014 also showed high homology to those detected in mainland Europe and Asia (Hanna et al., 2015). In December 2014, the novel H5N8 and reassortant H5N2 viruses were detected in wild birds in Washington, USA, and subsequently in backyard birds (Ip et al., 2015, 2016). Many of these birds were infected with the Asian-origin, H5N2, H5N8, and H5N1 HPAI viruses (Jhung and Nelson, 2015). In late November 2014, an outbreak of a novel HPAI H5N2 was reported in a turkey and chicken farm in Canada. Analysis suggested that five segments (PB2, PA, HA, M, and NS) of this virus were related to a Eurasian HPAI H5N8 and the remaining three segments were derived from North American lineage waterfowl viruses (Pasick et al., 2015).

Previous studies showed that the Glu627Lys, Asp701Asn, and Ser714Arg substitutions in the PB2 protein are associated with the host range and confer increased virulence of H5N1 viruses in mice (Shinya et al., 2004; Schat et al., 2012; Czudai-Matwich et al., 2014), while the Glu158Gly and Thr271Ala substitutions play key roles in enhanced polymerase activity of influenza viruses in mammalian host cells (Bussey et al., 2010; Zhou et al., 2011). Furthermore, a previous report suggested that the Thr97Ile substitution in the PA protein of H5 AIV plays a key role in enhanced virulence in mice and is implicated in the adaptation of AIVs to mammalian hosts (Song et al., 2009). In this study, none of these substitutions were observed in the PB2 or PA from the H5N2 viruses analyzed, suggesting that these H5N2 strains are low levels of pathogenicity for mice. The deletion of five amino acids “TIASV” (position 80–84) in the NS1 proteins of H5N1 AIVs has been observed with relatively high frequency in H5N1 AIVs, which might indicate adaptation to avian species (Long et al., 2008; Zhu et al., 2008). In this study, this deletion was only observed in the ZJ-514135, suggesting that this strain was more adaptive to birds than other three strains.

Waterfowl are known to harbor most of the AIV subtypes, and are considered a natural reservoir for these viruses. AIV infection is usually asymptomatic in domestic ducks, which provide a favorable environment for AIV reassortment (Kawaoka et al., 1988; Liu et al., 2003; Cardona et al., 2009). Our previous studies showed that reassortment events between H5 and other subtypes viruses occurred in domestic ducks in LPMs in Zhejiang Province, leading to the emergence of the novel H5N2, H5N6, and H5N8 viruses since 2013 (Wu et al., 2014a,b, 2015a). In this study, our results indicated that the H5 AIVs circulating in Eastern China had undergone extensive reassortment with other AIVs to create two genetic groups of viruses in poultry. Considering that the novel reassorted H5N2 viruses were isolated from poultry in this area, it is possible that these poultry play an important role in the generation of novel reassorted H5N2 AIVs. Thus, monitoring AIVs in migratory waterfowl and poultry has become a critical task in the prevention and control of avian influenza.

## Conclusion

Novel H5N2 AIVs were isolated from poultry in LPMs in Zhejiang Province, Eastern China, in 2015. Phylogenetic analysis suggested that these H5N2 viruses had undergone extensive reassortment to generate two genetic groups of viruses in poultry. These strains were found to be slightly virulent in mice, with the ability to replicate in mice without prior adaptation. As LPMs play an important role in the dissemination of AIVs, active surveillance to monitor novel AIVs in LPMs is required as an early warning system for AIV outbreaks.

## Author Contributions

HW and NW designed this study. HW, RL, XiuP, XiaP, LC, and FL performed the experiments and participated in the data collection and analysis. HW and NW drafted the manuscript.

## Conflict of Interest Statement

The authors declare that the research was conducted in the absence of any commercial or financial relationships that could be construed as a potential conflict of interest.

## Footnotes

1 http://www.ncbi.nlm.nih.gov
